# The “Art” of Ventricular Tachycardia Ablation

**DOI:** 10.19102/icrm.2018.090608

**Published:** 2018-06-15

**Authors:** Justin Hayase, Jason S. Bradfield

**Affiliations:** ^1^UCLA Cardiac Arrhythmia Center, Ronald Reagan UCLA Medical Center, Los Angeles, CA, USA

**Keywords:** Catheter ablation, electroanatomic mapping, ventricular tachycardia

High-volume ventricular tachycardia (VT) ablationists are often asked what their chosen “technique” is for VT ablation. Should VT be induced and mapped during tachycardia, or should a substrate-based approach be undertaken instead?

The inherent problem in this question is that every patient, every VT, and every scar are different. As physicians, we like to have clear “black or white” answers to clinical questions and to be able to state that the “right way” to do a procedure is “X”—in effect, do A, B, and C, and you will obtain the same result every time. However, the reality is that the difference between success and failure in a highly complex VT ablation may come down to experience and the willingness to think about all of the possible critical regions for a given VT or VTs, in a given scar, in a given substrate, in a given patient.

VT doesn’t always behave rationally. A VT that exits on the epicardial lateral left ventricle (LV) may have perfect pacemaps on the endocardial septal LV but have a critical isthmus in the midmyocardial anterior LV, for example.^1^ As Sohinki et al.^2^ note in this issue of *The Journal of Innovations in Cardiac Rhythm Management*, we electrophysiologists generally like to label VT origin by the 12-lead electocardiogram morphology. However, QRS morphology only informs us of the exit site, and ablation that is performed near an exit may not in fact yield lesions located anywhere near the critical isthmus in a large scar.

Regardless of whether mapping in VT or employing a purely substrate-based approach, without the performance of comprehensive mapping, critical regions may be missed. Sohinki et al.^2^ are correct when they state that right ventricular mapping is often overlooked but is essential for septal and inferior scars. Additional regions such as the periaortic valve area,^3^ intracavitary structures (papillary muscles), the coronary venous system,^4^ and the epicardium are frequently missed or disregarded.

Additionally, new techniques can often elucidate substrates not seen with standard voltage mapping. Isochronal late-activation mapping^5^ and alternate wavefronts^6^ are techniques that can be used to further delineate critical regions of VT circuits after comprehensive substrate mapping fails to localize the critical region and when the use of VT mapping is not feasible.

In the end, there is no “right way” to ablate VT. Often times, a combination of substrate and VT mapping is required. Comprehensive mapping, operator experience, and a thoughtful assessment of the data available can lead to improved outcomes.
